# Crystal structure, Hirshfeld surface analysis and DFT studies of (*E*)-4-methyl-2-{[(2-methyl-3-nitro­phen­yl)imino]­meth­yl}phenol

**DOI:** 10.1107/S2056989020011652

**Published:** 2020-09-04

**Authors:** Emine Berrin Cinar, Md. Serajul Haque Faizi, Nermin Kahveci Yagci, Onur Erman Dogan, Alev Sema Aydin, Erbil Agar, Necmi Dege, Ashraf Mashrai

**Affiliations:** a Ondokuz Mayıs University, Faculty of Arts and Sciences, Department of Physics, Samsun, Turkey; b Department of Chemistry, Langat Singh College, B.R.A. Bihar University, Muzaffarpur, Bihar-842001, India; c Kirikkale University, Department of Physics, Kirikkale 71450, Turkey; d Ondokuz Mayıs University, Faculty of Arts and Sciences, Department of, Chemistry, Samsun, Turkey; e Department of Pharmacy, University of Science and Technology, Ibb Branch, Ibb, Yemen

**Keywords:** crystal structure, 2-hy­droxy-5-methyl-benzaldehyde, 2-methyl-3-nitro-phenyl­amine, Schiff base

## Abstract

The title compound, C_15_H_14_N_2_O_3_, was prepared by condensation of 2-hy­droxy-5-methyl-benzaldehyde and 2-methyl-3-nitro-phenyl­amine in ethanol. The configuration of the C=N bond is *E*. An intra­molecular O—H⋯N hydrogen bond is present, forming an *S*(6) ring motif and inducing the phenol ring and the Schiff base to be nearly coplanar [C—C—N—C torsion angle of 178.53 (13)°]. In the crystal, mol­ecules are linked by C—H⋯O inter­actions, forming chains along the *b*-axis direction. The Hirshfeld surface analysis indicates that the most important contributions to the crystal packing are from H⋯H (37.2%), C⋯H (30.7%) and O⋯H (24.9%) inter­actions. The gas phase density functional theory (DFT) optimized structure at the B3LYP/ 6–311 G(d,p) level is compared to the experimentally determined mol­ecular structure in the solid state. The HOMO–LUMO behaviour was elucidated to determine the energy gap.

## Abstract 2

The title compound crystallizes with a single mol­ecule in the asymmetric unit. The phenol ring makes a dihedral angle of 36.56 (3)° with the nitro­benzene ring. In the crystal, mol­ecules are linked by C—H⋯O inter­actions, forming chains along the *b*-axis direction.

## Chemical context   

Over the past 25 years, extensive research has surrounded the synthesis and use of Schiff base compounds in organic and inorganic chemistry, as they have important medicinal and pharmaceutical applications. These compounds show biological activities including anti­bacterial, anti­fungal, anti­cancer and herbicidal activities (Desai *et al.*, 2001 ▸; Singh & Dash, 1988 ▸; Karia & Parsania, 1999 ▸). Schiff bases are also becoming increasingly important in the dye and plastics industries as well as for liquid-crystal technology and the mechanistic investigation of drugs used in pharmacology, biochemistry and physiology (Sheikhshoaie & Sharif, 2006 ▸). *ortho*-Hy­droxy Schiff base compounds such as the title compound can display two tautomeric forms, the enol–imine (OH) and keto–amine (NH) forms. Depending on the tautomers, two types of intra­molecular hydrogen bonds are generally observed in *ortho*-hy­droxy Schiff bases, namely, O—H⋯N in enol–imine and N—H⋯O in keto–amine tautomers (Tanak *et al.*, 2010 ▸). The present work is a part of an ongoing structural study of Schiff bases and their utilization in synthesis, their excited state proton-transfer properties and as fluorescent chemosensors (Faizi *et al.*, 2016 ▸, 2018 ▸; Kumar *et al.*, 2018 ▸; Mukherjee *et al.*, 2018 ▸). We report herein on the synthesis, crystal structure as well as Hirshfeld surface analysis of the title compound (I). The results of calculations by density functional theory (DFT) on (I) carried out at the B3LYP/6-311 G(d,p) level are compared with the experimentally determined mol­ecular structure in the solid state.
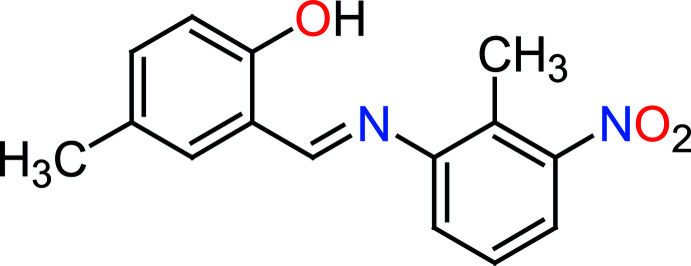


## Structural commentary   

**Figure 1:**
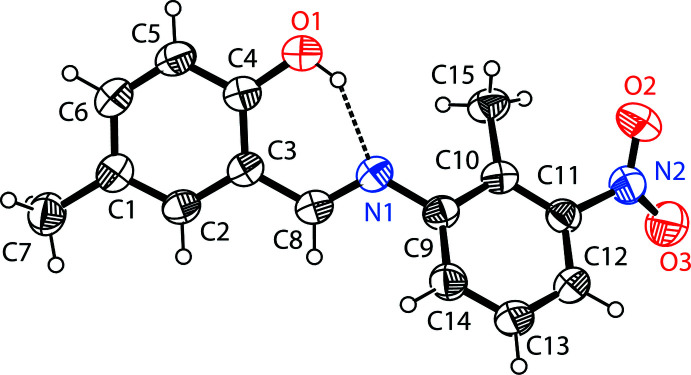
The mol­ecular structure of the title mol­ecule, with the atom-numbering scheme. Displacement ellipsoids are drawn at the 40% probability level. The intra­molecular O—H⋯N hydrogen bond (Table1) is shown as a dashed line.

The mol­ecular structure of the title compound, (I), is illustrated in Fig. 1 ▸. There is an intra­molecular O1—H1⋯N1 hydrogen bond (Table 1 ▸ and Fig. 1 ▸); this is a common feature also observed in related imine-phenol Schiff bases. It forms an *S*(6) ring motif and also induces the phenol ring and the Schiff base to be nearly coplanar, as indicated by the C3—C8—N1—C9 torsion angle of 178.53 (13)°. An intra­molecular C15—H15*B*⋯O2 inter­action is also observed. The phenol ring (C1–C8/O1) is inclined to the tolyl ring (C9–C14) by 37.57 (3)°, and the nitro group (N2/O2/O3) is inclined to the tolyl ring (C9—C14) by 35.05 (2)°. The configuration of the C8=N1 bond is *E*. The C4—O1 distance is 1.3455 (18) Å, which is close to normal values reported for single C—O bonds in phenols and salicyl­idene­amines (Ozeryanskii *et al.*, 2006 ▸). The N1—C8 bond is short at 1.2782 (19) Å, strongly indicating a C=N double bond, while the long C8—C3 bond [1.4486 (18) Å] implies a single bond. All of these data support the existence of the phenol–imine tautomer for (I) in the crystalline state. These features are similar to those observed in related 4-di­methyl­amino-*N*-salicylideneanilines (Pizzala *et al.*, 2000 ▸).

## Supra­molecular features   

**Figure 2:**
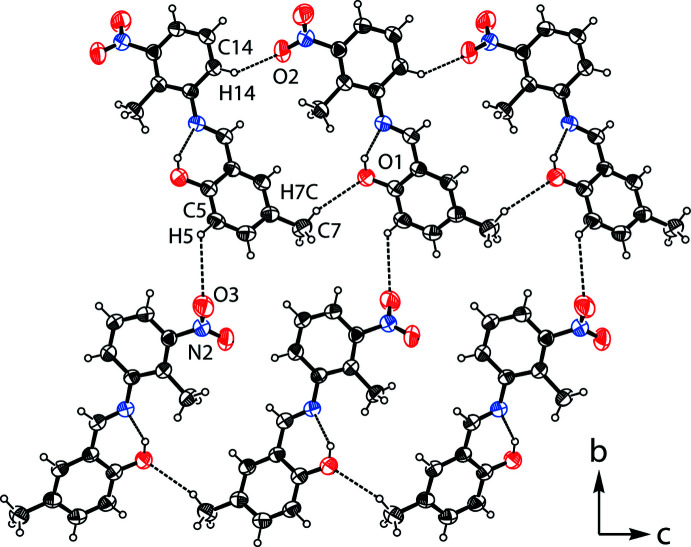
A view along the *a* axis of the chain formed by C—H⋯O inter­actions (dashed lines; see Table 1 ▸ for details).

In the crystal, mol­ecules are linked by two inter­molecular inter­actions, C14—H14⋯O2^i^ and C7—H7*C*⋯O1^i^, resulting in the formation of an infinite chain along the *b*-axis direction (Fig. 2 ▸ and Table 1 ▸).

## Hirshfeld surface analysis and two-dimensional fingerprint plots   

**Figure 3:**
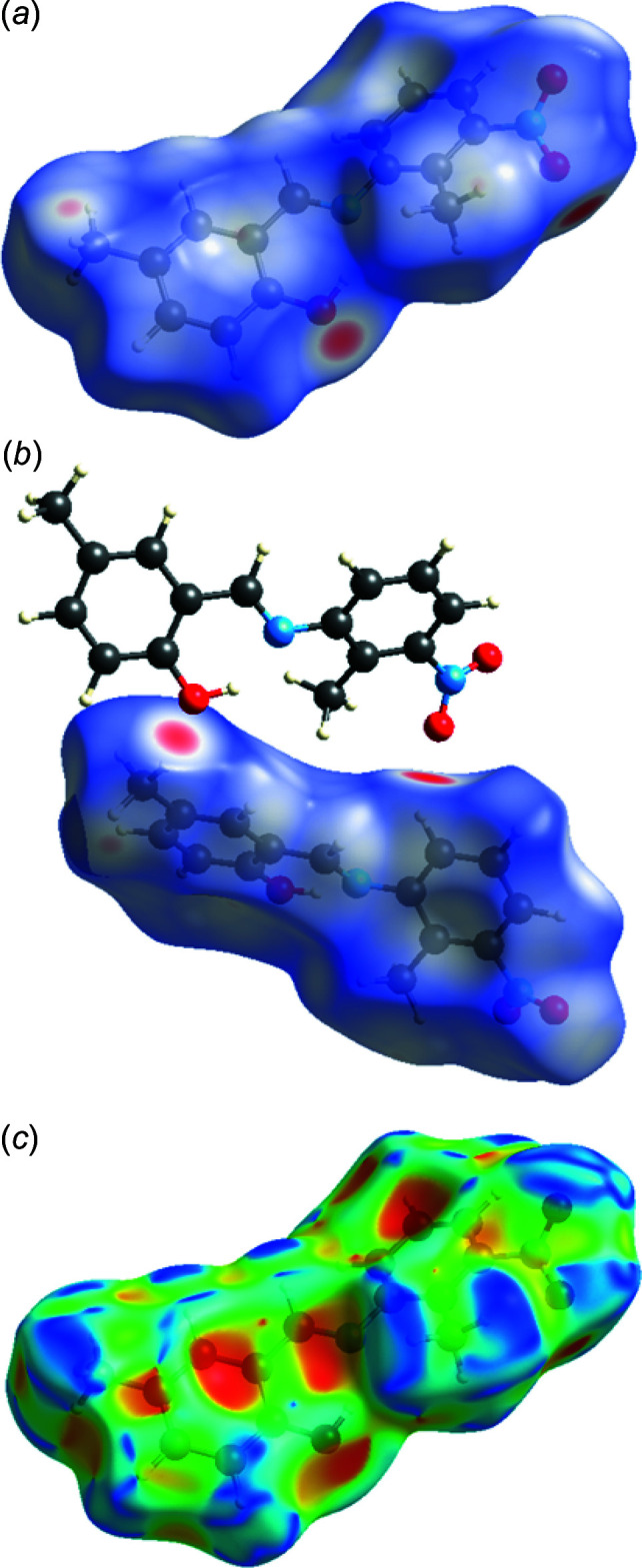
A view of the Hirshfeld surface mapped over (*a*) *d*_norm_ (*b*) C—H⋯O inter­actions and (*c*) shape-index.

**Figure 4:**
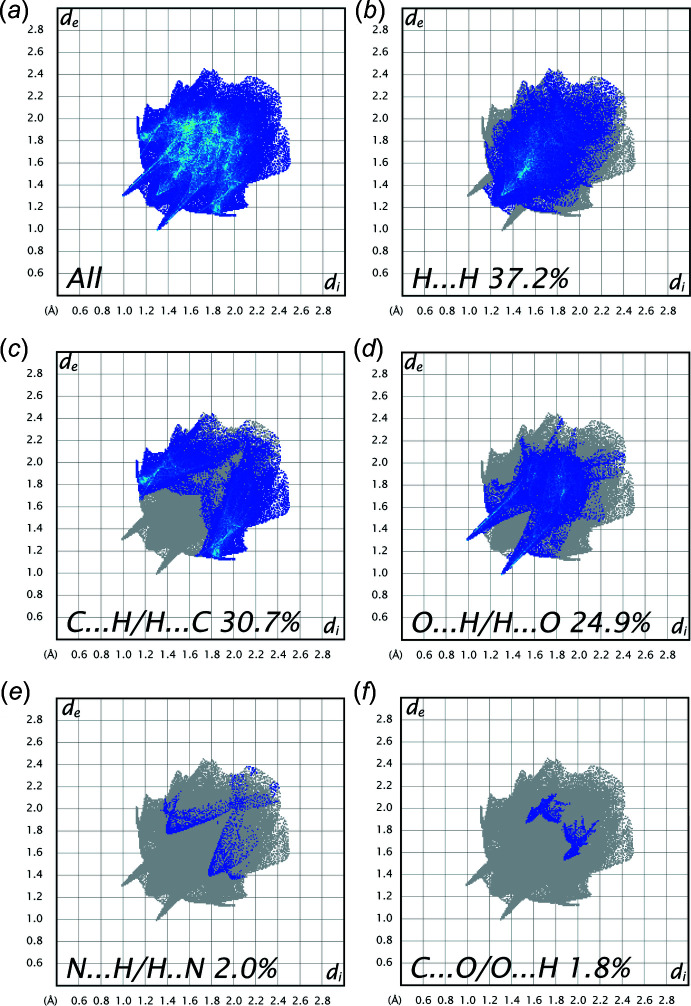
The overall two-dimensional fingerprint plot and those delineated into (*b*) H⋯H (37.2%), (*c*) C⋯H/H⋯C (30.7%), (*d*) O⋯H/H⋯O (24.9%), (*e*) N⋯H/H⋯N (2.0%) and (*f*) C⋯O/O⋯C (1.8%) contacts.

Hirshfeld surface analysis, together with two-dimensional fingerprint plots, is a powerful tool for the visualization and inter­pretation of inter­molecular contacts in mol­ecular crystals, since it provides a concise description of all inter­molecular inter­actions present in a crystal structure (Spackman & Jayatilaka, 2009 ▸; McKinnon *et al.*, 2007 ▸). All surfaces and fingerprint plots were generated using *CrystalExplorer3.1* (Turner *et al.*, 2017 ▸). The mappings of *d*_norm_ and shape-index for the title structure are shown in Fig. 3 ▸ *a* and 3*c*, respectively, with the prominent hydrogen-bonding inter­actions shown as intense red spots. The red colour indicates regions with shorter inter­molecular contacts, while blue colour shows regions with longer contact distance in the Hirshfeld surface. The darkest red spots on the Hirshfeld surface indicate contact points with atoms participating in inter­molecular C—H⋯O inter­actions that involve C14—H14 and the O2 of the nitro group (Table 1 ▸, Fig. 3 ▸ *b*). The two-dimensional fingerprint plots (Fig. 4 ▸ *a*–*f*) provide information about the percentage contributions of the various inter­atomic contacts. The most important are H⋯H inter­actions, which contribute 37.2% to the total Hirshfeld surface. Other contributions are from C⋯H (30.7%), O⋯H (24.9%), N⋯H (2.0%) and C⋯O (1.8%) contacts. There are also smaller contributions (not shown in Fig. 4 ▸) from O⋯O (1.7%), N⋯O (1.1%) and C⋯N (0.6%) contacts. The Hirshfeld surface analysis confirms the importance of H-atom contacts in establishing the packing. The large number of H⋯H and H⋯C inter­actions are induced dipole-dispersive (or van der Waals) inter­actions while O⋯H inter­actions are responsible for hydrogen bonds, which play important roles in the crystal packing (Hathwar *et al.*, 2015 ▸).

## DFT calculations   

**Figure 5:**
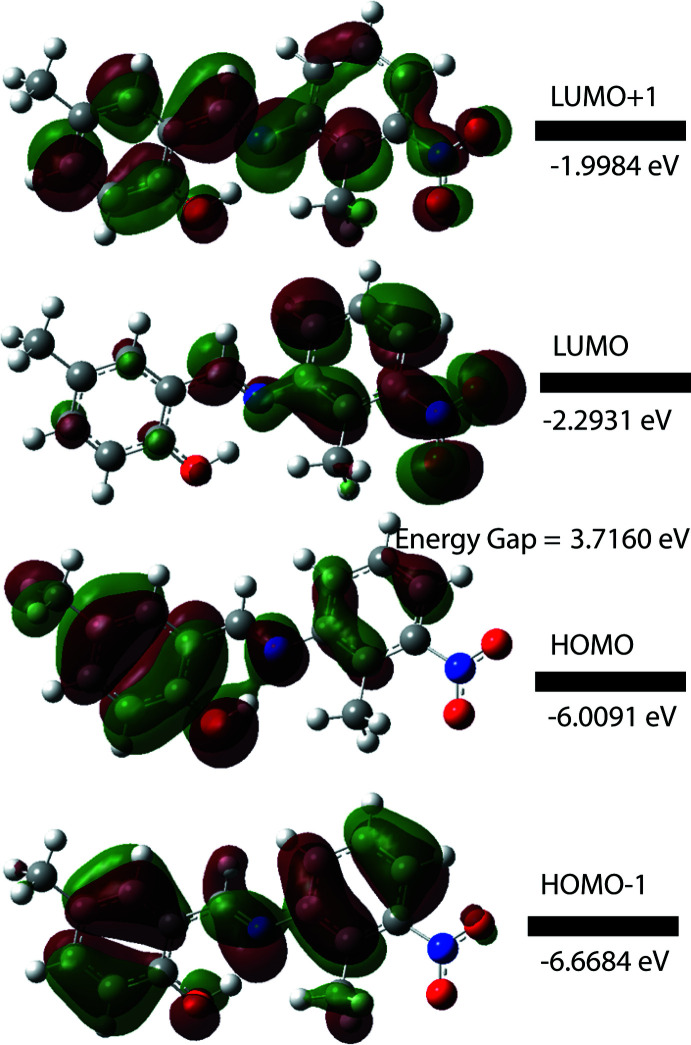
The energy band gap of the title compound.

The optimized structure of the title compound in the gas phase was generated theoretically *via* density functional theory (DFT) using the standard B3LYP functional and 6-311G(d,p) basis-set calculations (Becke, 1993 ▸) as implemented in *GAUSSIAN09* (Frisch *et al.*, 2009 ▸). The theoretical and experimental results are in good agreement (Table 2 ▸). The highest-occupied mol­ecular orbital (HOMO), acting as an electron donor, and the lowest-unoccupied mol­ecular orbital (LUMO), acting as an electron acceptor, are very important parameters for quantum chemistry. The electronic, optical and chemical reactivity properties of compounds are predicted by their frontier mol­ecular orbitals (Tanak, 2019 ▸). The HOMO–LUMO gap is used to analyse the chemical reactivity and stability of a mol­ecule. A mol­ecule with a small frontier orbital gap is more polarizable than one with a large gap and is considered a soft mol­ecule because of its high chemical reactivity and low kinetic stability. If the mol­ecule has a large HOMO–LUMO gap, the mol­ecule is more stable and less chemically reactive. The term ‘hard mol­ecule’ is used to describe such cases. The electron affinity (*A* = −*E*_HOMO_), the ionization potential (*I* = −*E*_LUMO_), HOMO–LUMO energy gap (Δ*E*), the chemical hardness (η) and softness (*S*) of the title compound were predicted based on the *E*_HOMO_ and *E*_LUMO_ energies. As a result of the large Δ*E* and η values (Table 3 ▸), the title compound can be classified as a hard mol­ecule. The electron distribution of the HOMO−1, HOMO, LUMO and the LUMO+1 energy levels for the title compound is shown in Fig. 5 ▸. The DFT study shows that HOMO and LUMO are localized in the plane extending from the whole 2-hy­droxy-5-methyl-benzaldehyde ring to the 2-methyl-3-nitro­phenyl­amine ring. The HOMO, HOMO−1 and LUMO+1 orbitals are delocalized over the two phenyl rings connected by the Schiff base bridge and HOMO and HOMO-1 can be said to be π-bonding orbitals. The LUMO orbital is delocalized on the 2-methyl-3-nitro­phenyl­amine ring and the C atom of the Schiff base. The LUMO and LUMO+1 orbitals exhibit π* anti­bonding character. The energy gap of (I) is 3.7160 eV, similar to that reported for the Schiff bases (*E*)-2-{[(3-chloro­phen­yl)imino]­meth­yl}-6-methyl­phenol (Δ*E* = 4.069 eV; Faizi *et al.*, 2019 ▸) and (*E*)-2-[(2-hy­droxy-5-meth­oxy­benzyl­idene)amino]­benzo­nitrile (Δ*E* = 3.520 eV; Saraçoğlu *et al.*, 2020 ▸).

## Database survey   

A search of the Cambridge Structural Database (CSD, version 5.39; Groom *et al.*, 2016 ▸) for the (*E*)-4-methyl-2-[(2-methyl-3-nitro-phenyl­imino)­meth­yl]phenol moiety resulted in no hits when both methyl groups were included in the search. Without the methyl groups, seven related compounds were found. Out of these, few are very similar to the title compound and some are metal complexes such as di­azido-[2,2′-{(4-nitro-1,2-phen­yl­­ene)bis­[(nitrilo)­methylyl­idene]}bis­(4-methyl­pheno­lato)]man­ganese (AGUGAN; Quan, 2018 ▸), where the ligand is similar to the title compound. There are two iron complexes, *viz*. {2-[({2-[bis­(3,5-di-*t*-butyl-2-oxybenz­yl)amino]-4,5-di­nitro­phen­yl}imino)­meth­yl]-4,6-di-*t*-butyl­phenolato}iron(III) meth­anol solvate hemihydrate (AROVIO; Wickramasinghe *et al.*, 2016 ▸) in which a *t*-butyl group is present and chloro-{2,4-di-*t*-butyl-6-[({2-[(3,5-di-*t*-butyl-2-oxybenzyl­idene)amino]-4,5-di­nitro­phen­yl}imino)­meth­yl]phenolato}iron(III) (AROVOU; Wickramasinghe *et al.*, 2016 ▸) in which two nitro groups are attached to one aromatic ring. A nickel complex [*N*,*N*′-(4,5-di­nitro-1,2-phenyl­ene)bis­(3,5-di-*t*-butyl­salicylaldiminato)]nickel(II) methanol solvate (BOQPAZ; Rotthaus *et al.*, 2009 ▸) and a cobalt complex with a similar ligand {2,2′-[{[2-({[3,5-di-*t*-butyl-2-oxyphen­yl]methyl­idene}amino)-4,5-di­nitro­phen­yl]aza­nedi­yl}bis­(methyl­ene)]bis­(4,6-di-*t*-butyl­phenolato)}meth­ano­lcobalt(III) methanol solvate (FORJOO; Basu *et al.*, 2019 ▸) have also been reported. The compound most analogous to the title compound is *N*-(3,5-di-*t*-butyl­salicyl­idene)-3-nitro­aniline (KIPMEB; Harada *et al.*, 1999 ▸; KIPMEB03; Koshima *et al.*, 2011 ▸) in which a *t*-butyl group is present. In all of the above structures except AGUGAN, both methyl groups are absent and this structure is the most similar to the title compound.

## Synthesis and crystallization   

The title compound was prepared by refluxing mixed solutions of 2-hy­droxy-5-methyl-benzaldehyde (38.0 mg, 0.28 mmol) in ethanol (15 ml) and 2-methyl-3-nitro-phenyl­amine (42.0 mg, 0.28 mmol) in ethanol (15 ml). The reaction mixture was stirred for 5 h under reflux. Single crystals of the title compound suitable for X-ray analysis were obtained by slow evaporation of an ethanol solution (yield 65%, yellow prisms, m.p. 410–412 K).

## Refinement   

Crystal data, data collection and structure refinement details are summarized in Table 4 ▸. The hy­droxy H atom was located in a difference-Fourier map and positional parameters were refined freely, *U*_iso_(H) = 1.5*U*_eq_(O). Other H atoms were fixed geometrically and treated as riding with C—H = 0.96 Å (meth­yl) or 0.93 Å (aromatic), *U*_iso_(H) = 1.2*U*_eq_(C) or 1.5*U*_eq_(Cmeth­yl).

## Supplementary Material

Crystal structure: contains datablock(s) I. DOI: 10.1107/S2056989020011652/zl2794sup1.cif

Structure factors: contains datablock(s) I. DOI: 10.1107/S2056989020011652/zl2794Isup2.hkl

Supporting information file. DOI: 10.1107/S2056989020011652/zl2794Isup3.cml

CCDC reference: 2025323

Additional supporting information:  crystallographic information; 3D view; checkCIF report

## Figures and Tables

**Table 1:** Hydrogen-bond geometry (Å, °)

*D*—H⋯*A*	*D*—H	H⋯*A*	*D*⋯*A*	*D*—H⋯*A*
C7—H7*C*⋯O1^i^	0.96	2.54	3.468 (2)	163
C14—H14⋯O2^i^	0.93	2.40	3.2064 (19)	145
C15—H15*B*⋯O2	0.96	2.33	2.840 (2)	113
O1—H1⋯N1	0.95 (3)	1.78 (3)	2.6032 (16)	143 (3)

Symmetry code: (i) 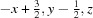.

**Table 2:** Comparison of selected observed (X-ray data) and calculated (DFT) geometric parameters (Å, °)

Parameter	X-ray	B3LYP/6–311G(d,p)
O1—C4	1.3455 (18)	1.3406
N1—C8	1.2782 (19)	1.2946
N2—C11	1.4728 (19)	1.4763
C9—N1	1.4169 (17)	1.4080
C8—C3	1.4486 (18)	1.4457
N1—C8—C3	121.84 (13)	122.41
C8—N1—C9	120.92 (12)	120.91
O2—N2—O3	122.32 (14)	124.17

**Table 3:** The energy band gap of the title compound

Mol­ecular Energy, (eV)	Compound (I)
Total Energy, *TE* (eV)	−24894.6063
*E*_HOMO_ (eV)	−6.0091
*E*_LUMO_ (eV)	−2.2931
Gap, *ΔE* (eV)	3.7160
Dipole moment, *μ* (Debye)	6.545
Ionization potential, *I* (eV)	6.009
Electron affinity, *A*	2.293
Electronegativity, *χ*	4.151
Hardness, *η*	1.858
Electrophilicity index, *ω*	4.636
Softness, *σ*	0.269
Fraction of electron transferred, *ΔN*	0.744

**Table 4:** Experimental details

Crystal data	
Chemical formula	C_15_H_14_N_2_O_3_
*M*_r_	270.28
Crystal system, space group	Orthorhombic, *P* *b* *c* *a*
Temperature (K)	296
*a*, *b*, *c* (Å)	7.3925 (3), 15.4082 (6), 23.5750 (9)
*V* (Å^3^)	2685.31 (18)
*Z*	8
Radiation type	Mo *K*α
μ (mm^−1^)	0.10
Crystal size (mm)	0.72 × 0.66 × 0.59

Data collection	
Diffractometer	Stoe IPDS 2
Absorption correction	Integration (*X-RED32*; Stoe & Cie, 2002 ▸)
*T*_min_, *T*_max_	0.935, 0.968
No. of measured, independent and observed [*I* > 2σ(*I*)] reflections	18040, 3618, 2258
*R*_int_	0.035
(sin θ/λ)_max_ (Å^−1^)	0.686

Refinement	
*R*[*F* ^2^ > 2σ(*F* ^2^)], *wR*(*F* ^2^), *S*	0.043, 0.125, 1.04
No. of reflections	3618
No. of parameters	187
H-atom treatment	H atoms treated by a mixture of independent and constrained refinement
Δρ_max_, Δρ_min_ (e Å^−3^)	0.15, −0.13

Computer programs: *X-AREA*, *X-RED32* and *X-SHAPE* (Stoe & Cie, 2002 ▸), *SHELXT2014/5* (Sheldrick, 2015*a* ▸), *SHELXL2018/3* (Sheldrick, 2015*b* ▸), *ORTEP-3 for Windows* (Farrugia, 2012 ▸), *PLATON* (Spek, 2020 ▸), *publCIF* (Westrip, 2010 ▸) and *Mercury* (Macrae *et al.*, 2020 ▸).

## supplementary crystallographic information

### Crystal data

**Table:**

C_15_H_14_N_2_O_3_	*D*_x_ = 1.337 Mg m^−^^3^
*M**_r_* = 270.28	Mo *K*α radiation, λ = 0.71073 Å
Orthorhombic, *P**b**c**a*	Cell parameters from 15516 reflections
*a* = 7.3925 (3) Å	θ = 1.6–29.6°
*b* = 15.4082 (6) Å	µ = 0.10 mm^−^^1^
*c* = 23.5750 (9) Å	*T* = 296 K
*V* = 2685.31 (18) Å^3^	Prism, yellow
*Z* = 8	0.72 × 0.66 × 0.59 mm
*F*(000) = 1136	

### Data collection

**Table:**

STOE IPDS 2 diffractometer	3618 independent reflections
Radiation source: sealed X-ray tube, 12 x 0.4 mm long-fine focus	2258 reflections with *I* > 2σ(*I*)
Plane graphite monochromator	*R*_int_ = 0.035
Detector resolution: 6.67 pixels mm^-1^	θ_max_ = 29.2°, θ_min_ = 1.7°
rotation method scans	*h* = −8→10
Absorption correction: integration (X-RED32; Stoe & Cie, 2002)	*k* = −19→20
*T*_min_ = 0.935, *T*_max_ = 0.968	*l* = −32→32
18040 measured reflections	

### Refinement

**Table:**

Refinement on *F*^2^	Hydrogen site location: mixed
Least-squares matrix: full	H atoms treated by a mixture of independent and constrained refinement
*R*[*F*^2^ > 2σ(*F*^2^)] = 0.043	*w* = 1/[σ^2^(*F*_o_^2^) + (0.0659*P*)^2^] where *P* = (*F*_o_^2^ + 2*F*_c_^2^)/3
*wR*(*F*^2^) = 0.125	(Δ/σ)_max_ = 0.001
*S* = 1.03	Δρ_max_ = 0.14 e Å^−^^3^
3618 reflections	Δρ_min_ = −0.13 e Å^−^^3^
187 parameters	Extinction correction: SHELXL2018/3 (Sheldrick, 2015b), Fc^*^=kFc[1+0.001xFc^2^λ^3^/sin(2θ)]^-1/4^
0 restraints	Extinction coefficient: 0.0039 (10)

### Special details

**Table:**

Geometry. All esds (except the esd in the dihedral angle between two l.s. planes) are estimated using the full covariance matrix. The cell esds are taken into account individually in the estimation of esds in distances, angles and torsion angles; correlations between esds in cell parameters are only used when they are defined by crystal symmetry. An approximate (isotropic) treatment of cell esds is used for estimating esds involving l.s. planes.

### Fractional atomic coordinates and isotropic or equivalent isotropic displacement parameters (Å^2^)

**Table:**

	*x*	*y*	*z*	*U*_iso_*/*U*_eq_	
C1	0.74349 (19)	0.20861 (10)	0.60276 (6)	0.0596 (4)	
C2	0.70581 (18)	0.23031 (10)	0.54705 (6)	0.0559 (3)	
H2	0.646600	0.189998	0.524307	0.067*	
C3	0.75324 (18)	0.31034 (9)	0.52368 (5)	0.0519 (3)	
C4	0.8454 (2)	0.37053 (10)	0.55780 (6)	0.0577 (3)	
C5	0.8823 (2)	0.35005 (11)	0.61387 (6)	0.0665 (4)	
H5	0.942132	0.389899	0.636800	0.080*	
C6	0.8309 (2)	0.27125 (11)	0.63571 (6)	0.0650 (4)	
H6	0.855030	0.259165	0.673614	0.078*	
C7	0.6923 (3)	0.12209 (12)	0.62715 (7)	0.0773 (5)	
H7A	0.592310	0.129208	0.652729	0.116*	
H7B	0.793548	0.098209	0.647305	0.116*	
H7C	0.658178	0.083481	0.597033	0.116*	
C8	0.70208 (18)	0.33130 (10)	0.46597 (5)	0.0544 (3)	
H8	0.640422	0.290150	0.444545	0.065*	
C9	0.69171 (18)	0.42321 (9)	0.38670 (5)	0.0525 (3)	
C10	0.64261 (17)	0.50918 (9)	0.37357 (5)	0.0515 (3)	
C11	0.60096 (19)	0.52470 (9)	0.31696 (6)	0.0546 (3)	
C12	0.6044 (2)	0.46167 (10)	0.27547 (6)	0.0668 (4)	
H12	0.571736	0.474947	0.238372	0.080*	
C13	0.6565 (3)	0.37947 (10)	0.28964 (6)	0.0723 (5)	
H13	0.661926	0.336515	0.261979	0.087*	
C14	0.7012 (2)	0.36023 (10)	0.34503 (6)	0.0630 (4)	
H14	0.738019	0.304345	0.354450	0.076*	
C15	0.6285 (2)	0.57517 (11)	0.42042 (6)	0.0680 (4)	
H15A	0.580390	0.548033	0.453817	0.102*	
H15B	0.549860	0.621413	0.408760	0.102*	
H15C	0.746420	0.598158	0.428556	0.102*	
N1	0.73931 (16)	0.40478 (8)	0.44366 (5)	0.0563 (3)	
N2	0.55057 (19)	0.61224 (8)	0.29747 (6)	0.0675 (3)	
O1	0.89814 (18)	0.44842 (7)	0.53800 (5)	0.0778 (4)	
O2	0.6215 (2)	0.67456 (8)	0.31898 (6)	0.0945 (4)	
O3	0.4416 (2)	0.61880 (9)	0.25937 (6)	0.1073 (5)	
H1	0.864 (4)	0.4546 (18)	0.4992 (14)	0.161*	

### Atomic displacement parameters (Å^2^)

**Table:**

	*U*^11^	*U*^22^	*U*^33^	*U*^12^	*U*^13^	*U*^23^
C1	0.0528 (8)	0.0715 (9)	0.0545 (7)	0.0031 (7)	0.0025 (6)	0.0003 (6)
C2	0.0493 (7)	0.0632 (9)	0.0551 (7)	−0.0006 (6)	−0.0014 (5)	−0.0070 (6)
C3	0.0469 (7)	0.0589 (8)	0.0500 (6)	0.0036 (6)	0.0001 (5)	−0.0051 (6)
C4	0.0574 (8)	0.0598 (9)	0.0559 (7)	−0.0002 (6)	−0.0020 (6)	−0.0057 (6)
C5	0.0723 (10)	0.0711 (10)	0.0562 (8)	−0.0027 (8)	−0.0083 (7)	−0.0112 (7)
C6	0.0647 (9)	0.0796 (11)	0.0506 (7)	0.0056 (8)	−0.0038 (6)	−0.0021 (7)
C7	0.0793 (11)	0.0836 (12)	0.0690 (10)	−0.0088 (9)	−0.0052 (8)	0.0130 (8)
C8	0.0489 (7)	0.0604 (9)	0.0540 (7)	0.0006 (6)	−0.0003 (6)	−0.0071 (6)
C9	0.0496 (7)	0.0577 (8)	0.0501 (6)	−0.0001 (6)	0.0031 (5)	−0.0052 (6)
C10	0.0443 (7)	0.0561 (8)	0.0542 (7)	−0.0011 (6)	0.0020 (5)	−0.0075 (6)
C11	0.0551 (8)	0.0520 (8)	0.0567 (7)	−0.0036 (6)	0.0031 (6)	−0.0013 (6)
C12	0.0860 (11)	0.0665 (9)	0.0480 (7)	−0.0063 (8)	0.0029 (7)	−0.0029 (6)
C13	0.1020 (13)	0.0614 (9)	0.0535 (8)	−0.0023 (8)	0.0097 (8)	−0.0112 (7)
C14	0.0770 (10)	0.0534 (8)	0.0585 (8)	0.0046 (7)	0.0071 (7)	−0.0039 (6)
C15	0.0735 (10)	0.0661 (9)	0.0644 (8)	0.0095 (8)	−0.0050 (7)	−0.0177 (7)
N1	0.0543 (6)	0.0622 (7)	0.0523 (6)	0.0026 (5)	−0.0007 (5)	−0.0029 (5)
N2	0.0747 (9)	0.0625 (8)	0.0654 (7)	−0.0006 (7)	0.0020 (6)	0.0020 (6)
O1	0.0998 (9)	0.0647 (7)	0.0687 (6)	−0.0177 (6)	−0.0139 (6)	−0.0025 (5)
O2	0.1208 (11)	0.0561 (7)	0.1065 (9)	−0.0115 (7)	−0.0104 (8)	−0.0056 (6)
O3	0.1330 (13)	0.0889 (9)	0.1001 (9)	0.0089 (9)	−0.0457 (9)	0.0120 (7)

### Geometric parameters (Å, º)

**Table:**

C1—C2	1.3836 (19)	C9—C10	1.4079 (19)
C1—C6	1.397 (2)	C9—N1	1.4169 (17)
C1—C7	1.501 (2)	C10—C11	1.3904 (19)
C2—C3	1.395 (2)	C10—C15	1.5048 (19)
C2—H2	0.9300	C11—C12	1.3787 (19)
C3—C4	1.4039 (19)	C11—N2	1.4728 (19)
C3—C8	1.4486 (18)	C12—C13	1.365 (2)
C4—O1	1.3455 (18)	C12—H12	0.9300
C4—C5	1.386 (2)	C13—C14	1.379 (2)
C5—C6	1.372 (2)	C13—H13	0.9300
C5—H5	0.9300	C14—H14	0.9300
C6—H6	0.9300	C15—H15A	0.9600
C7—H7A	0.9600	C15—H15B	0.9600
C7—H7B	0.9600	C15—H15C	0.9600
C7—H7C	0.9600	N2—O2	1.2057 (17)
C8—N1	1.2782 (19)	N2—O3	1.2109 (18)
C8—H8	0.9300	O1—H1	0.95 (3)
C9—C14	1.3826 (19)		
			
C2—C1—C6	117.00 (14)	C14—C9—N1	121.34 (13)
C2—C1—C7	121.84 (14)	C10—C9—N1	117.45 (11)
C6—C1—C7	121.16 (13)	C11—C10—C9	115.47 (12)
C1—C2—C3	122.53 (13)	C11—C10—C15	124.95 (13)
C1—C2—H2	118.7	C9—C10—C15	119.48 (12)
C3—C2—H2	118.7	C12—C11—C10	123.76 (13)
C2—C3—C4	118.64 (12)	C12—C11—N2	115.37 (12)
C2—C3—C8	120.15 (12)	C10—C11—N2	120.86 (12)
C4—C3—C8	121.17 (13)	C13—C12—C11	119.01 (14)
O1—C4—C5	118.48 (13)	C13—C12—H12	120.5
O1—C4—C3	122.08 (13)	C11—C12—H12	120.5
C5—C4—C3	119.45 (14)	C12—C13—C14	119.91 (14)
C6—C5—C4	120.31 (14)	C12—C13—H13	120.0
C6—C5—H5	119.8	C14—C13—H13	120.0
C4—C5—H5	119.8	C13—C14—C9	120.65 (14)
C5—C6—C1	122.04 (13)	C13—C14—H14	119.7
C5—C6—H6	119.0	C9—C14—H14	119.7
C1—C6—H6	119.0	C10—C15—H15A	109.5
C1—C7—H7A	109.5	C10—C15—H15B	109.5
C1—C7—H7B	109.5	H15A—C15—H15B	109.5
H7A—C7—H7B	109.5	C10—C15—H15C	109.5
C1—C7—H7C	109.5	H15A—C15—H15C	109.5
H7A—C7—H7C	109.5	H15B—C15—H15C	109.5
H7B—C7—H7C	109.5	C8—N1—C9	120.92 (12)
N1—C8—C3	121.84 (13)	O2—N2—O3	122.32 (14)
N1—C8—H8	119.1	O2—N2—C11	119.22 (13)
C3—C8—H8	119.1	O3—N2—C11	118.44 (13)
C14—C9—C10	121.14 (12)	C4—O1—H1	110.3 (17)
			
C6—C1—C2—C3	0.6 (2)	N1—C9—C10—C15	−4.75 (19)
C7—C1—C2—C3	−179.72 (14)	C9—C10—C11—C12	0.6 (2)
C1—C2—C3—C4	1.1 (2)	C15—C10—C11—C12	−175.87 (15)
C1—C2—C3—C8	−176.78 (13)	C9—C10—C11—N2	−178.94 (12)
C2—C3—C4—O1	178.89 (13)	C15—C10—C11—N2	4.6 (2)
C8—C3—C4—O1	−3.3 (2)	C10—C11—C12—C13	−2.0 (2)
C2—C3—C4—C5	−1.7 (2)	N2—C11—C12—C13	177.53 (15)
C8—C3—C4—C5	176.10 (14)	C11—C12—C13—C14	1.3 (3)
O1—C4—C5—C6	−179.93 (14)	C12—C13—C14—C9	0.8 (3)
C3—C4—C5—C6	0.7 (2)	C10—C9—C14—C13	−2.3 (2)
C4—C5—C6—C1	1.1 (2)	N1—C9—C14—C13	−179.18 (14)
C2—C1—C6—C5	−1.8 (2)	C3—C8—N1—C9	178.53 (12)
C7—C1—C6—C5	178.60 (15)	C14—C9—N1—C8	−36.9 (2)
C2—C3—C8—N1	178.24 (13)	C10—C9—N1—C8	146.03 (13)
C4—C3—C8—N1	0.4 (2)	C12—C11—N2—O2	−144.51 (15)
C14—C9—C10—C11	1.5 (2)	C10—C11—N2—O2	35.0 (2)
N1—C9—C10—C11	178.59 (12)	C12—C11—N2—O3	33.9 (2)
C14—C9—C10—C15	178.20 (14)	C10—C11—N2—O3	−146.51 (16)

### Hydrogen-bond geometry (Å, º)

**Table:**

*D*—H···*A*	*D*—H	H···*A*	*D*···*A*	*D*—H···*A*
C7—H7*C*···O1^i^	0.96	2.54	3.468 (2)	163
C14—H14···O2^i^	0.93	2.40	3.2064 (19)	145
C15—H15*B*···O2	0.96	2.33	2.840 (2)	113
O1—H1···N1	0.95 (3)	1.78 (3)	2.6032 (16)	143 (3)

Symmetry code: (i) −*x*+3/2, *y*−1/2, *z*.
